# Effects of transcranial direct current stimulation on motor skills learning in healthy adults through the activation of different brain regions: A systematic review

**DOI:** 10.3389/fnhum.2022.1021375

**Published:** 2022-10-06

**Authors:** Shuo Qi, Zhiqiang Liang, Zhen Wei, Yu Liu, Xiaohui Wang

**Affiliations:** School of Sport and Health, Shanghai University of Sport, Shanghai, China

**Keywords:** transcranial direct current stimulation, motor skills learning, primary motor cortex, cerebellum, dorsolateral prefrontal cortex, neural circuitry

## Abstract

**Objective:**

This systematic review aims to analyze existing literature of the effects of transcranial direct current stimulation (tDCS) on motor skills learning of healthy adults and discuss the underlying neurophysiological mechanism that influences motor skills learning.

**Methods:**

This systematic review has followed the recommendations of the Preferred Reporting Items for Systematic reviews and Meta-Analyses. The PubMed, EBSCO, and Web of Science databases were systematically searched for relevant studies that were published from database inception to May 2022. Studies were included based on the Participants, Intervention, Comparison, Outcomes, and Setting inclusion strategy. The risk of bias was evaluated by using the Review manager 5.4 tool. The quality of each study was assessed with the Physiotherapy Evidence Database (PEDro) scale.

**Results:**

The electronic search produced 142 studies. Only 11 studies were included after filtering. These studies performed well in terms of distribution, blinding availability and selective reporting. They reported that tDCS significantly improved motor skills learning. The main outcomes measure were the improvement of the motor sequence tasks and specific motor skills. Nine studies showed that tDCS interventions reduced reaction time to complete motor sequence tasks in healthy adults and two studies showed that tDCS interventions improved golf putting task performance.

**Conclusion:**

The included studies showed that tDCS can help healthy adults to improve the motor skills learning by activating different brain regions, such as the primary motor cortex, left dorsolateral prefrontal cortex and right cerebellum. However, the number of included studies was limited, and the sample sizes were small. Therefore, more studies are urgently needed to validate the results of current studies and further explore the underlying neurophysiological mechanisms of tDCS in the future.

## Introduction

Transcranial direct current stimulation (tDCS) is a non-invasive neuromodulation technique that uses a stable, low-intensity direct current (1-2 mA) to regulate the activity of cortical neurons (Nitsche et al., 2008). The components of tDCS consist of a battery-powered stimulator and two or more electrodes (anode and cathode) placed on the scalp (Jamil et al., 2017). The current passes through the scalp, through the outer layer of the cortex, and then reaches the cortex, where it regulates the membrane polarity of neurons in a certain area under the neural tissue of the cerebral cortex (Fertonani and Miniussi, 2017). Subthreshold stimulation provided by tDCS can depolarize or hyperpolarize the resting membrane potential of neurons, which depends on the stimulation parameters of tDCS and the neuron direction related to the induced electric field (Sudbrack-Oliveira et al., 2021). tDCS is non-invasive, efficient, simple to operate and inexpensive with numerous applications in various fields. Anodal tDCS (a-tDCS) usually aims to increase the excitability of the targeted cortical regions by depolarizing the membrane potential of neurons, whereas cathodal tDCS (c-tDCS) frequently inhibits the neural excitability of the targeted brain regions (Nitsche et al., 2003a).

Motor skills are needed for daily life activities. They are the ability to complete target actions during human movement. Motor skills learning is a process in which the human body receives various signal stimuli and establishes complex conditioned reflexes under the guidance of the cerebral cortex. It is also a process of establishing a balance between excitation and inhibition in the cerebral cortex (Shmuelof et al., 2012). Motor skills learning adaptation is related to the functional connectivity of the brain network, which is defined as a dependency relationship reflecting the degree of non-directional synchronization between two brain regions (Polanía et al., 2011), particularly brain regions, such as the primary motor cortex (M1), cerebellum, supplementary motor area (SMA) and dorsolateral prefrontal cortex (DLPFC) (Landi et al., 2011). Studies have demonstrated that tDCS can increase synaptic plasticity and enhance the functional connection of premotor, motor and sensorimotor regions (Kuo et al., 2014). The above mechanisms have a positive effect on brain activities in motor skills learning and promote motor skills learning. In recent years, researchers have investigated the effect of tDCS on the exercise performance of healthy individuals, and found that tDCS can improve a variety of physical functions, including muscle fatigue (Angius et al., 2016), motor skills learning (Ehsani et al., 2016), motor sensation (Olma et al., 2013), balance control (Saruco et al., 2017) and muscle strength (Abdelmoula et al., 2016). When a-tDCS acts on the M1 region, it can significantly increase the performance level of motor learning and promote the acquisition and maintenance of motor skills (Nitsche et al., 2003b). C-tDCS can also improve performance in motor learning and golf putting practice by acting on the left DLPFC region (Zhu et al., 2015). Given the effective impact of tDCS on motor skills learning, a systematic review of published studies could provide valuable summaries of the effects of tDCS on motor skills learning.

Therefore, this study aims to review systematically the peer-reviewed publications available to date on the effects of tDCS on motor skills learning in healthy adults, then discusses its underlying neurophysiological mechanisms. This review provides an improved understanding of the research work on this topic and will eventually help optimize the application of tDCS in promoting motor skills learning in the future.

## Methods

The method of this review was developed under the recommendations of the Preferred Reporting Items for Systematic Reviews and Meta-Analyses and the Cochrane Handbook for Systematic Reviews of Interventions (Cumpston et al., 2019).

### Search strategy

In this systematic review, the three databases of PubMed, Web of Science and EBSCO were comprehensively searched up to May 2022. The phrases “motor skills,” “motor learning” and “memory” were separately combined with the phrases “transcranial direct current stimulation” or “tDCS” in all databases. The Boolean operators “AND” and “OR” were used to combine keywords based on the recommendations of each database. All search results were imported into the EndNote reference manager (EndNote X9, USA, Stanford) to collect and find duplicate records automatically.

### Eligibility criteria and article selection

Studies were included following the inclusion criteria of the Participants, Intervention, Comparison, Outcomes, and Study design.

**Figure 1:**
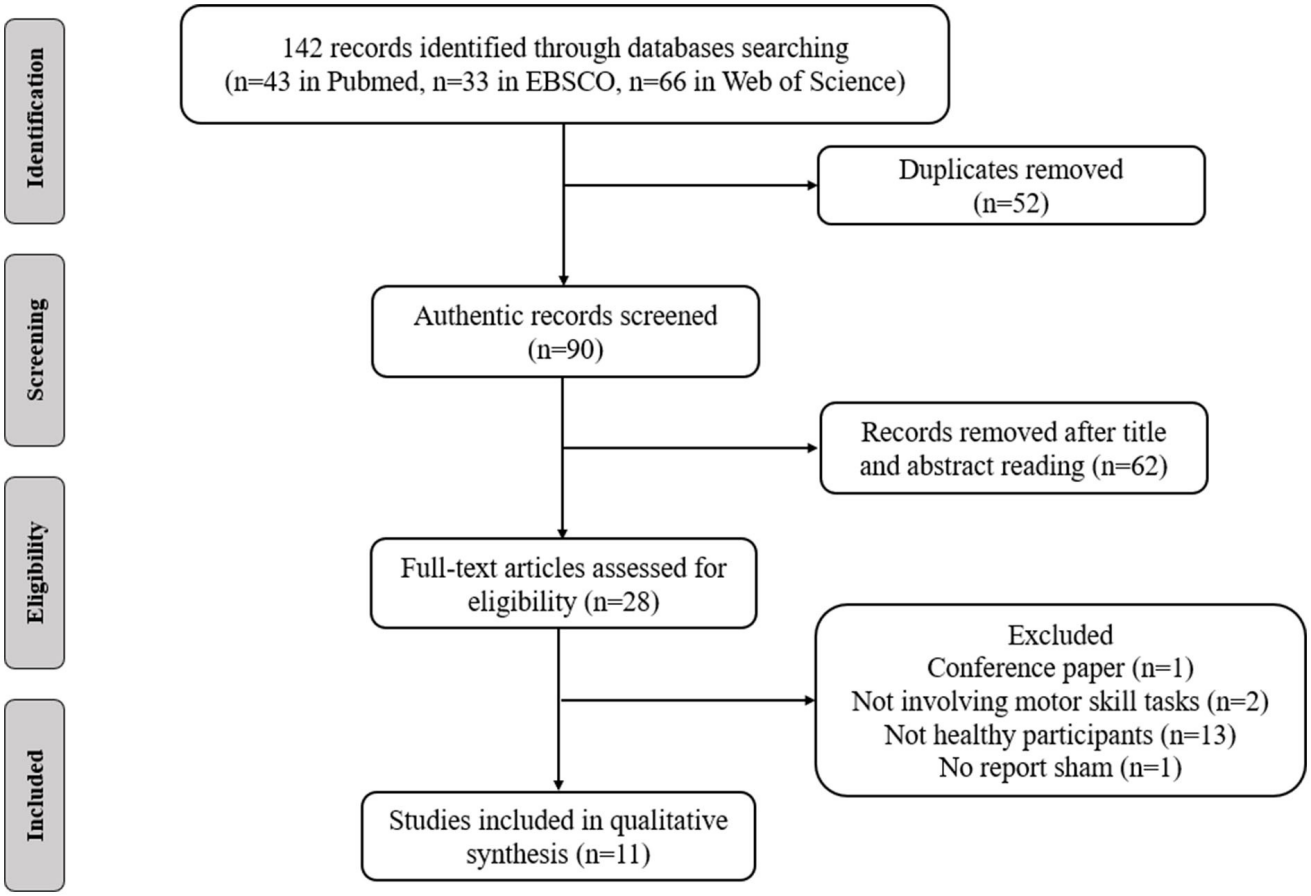
Flow diagram of the search strategy.

1. Participants: The participants were healthy adults without a history of musculoskeletal injury and obvious neurological diseases. The age range of healthy adults is 18–56 years old.
2. Intervention: The intervention method was tDCS regardless of stimulation type, intensity, duration or electrode position.
3. Comparison: The comparison was with sham tDCS.
4. Outcomes: The main outcomes measure were the improvement of the motor sequence tasks and specific motor skills, such as serial reaction time task (SRTT), sequential finger tapping tasks (SFTT), sequential visual isometric pinch task (SVIPT), and golf putting tasks. Improvements in motor skills learning are assessed through sequential task reaction times and golf putting scores.
5. Study design: The study included randomized, crossover, and sham controlled designs. Animal studies and non-English studies were excluded. Comments, case reports, letters, opinions and conference abstracts were also removed (Figure 1).

Two researchers independently examined the search results and resolved differences through discussion (QS and ZL). The abstracts and full texts of relevant articles were read carefully and only those that qualified were selected. Then, the researcher further confirmed the selected articles and discussed possible disagreements. If disagreements remained, a fifth researcher was consulted, and the results were evaluated (XW).

### Data extraction

Microsoft Excel (Microsoft Corporation, Redmond, WA, USA) was used to summarize the original data from the included articles. The author, sample, age, anodal/cathodal location, current intensity, electrode size, current density, duration, work tasks, and main outcome measures were all summarized.

### Quality and risk-of-bias assessments

**Figure 2:**
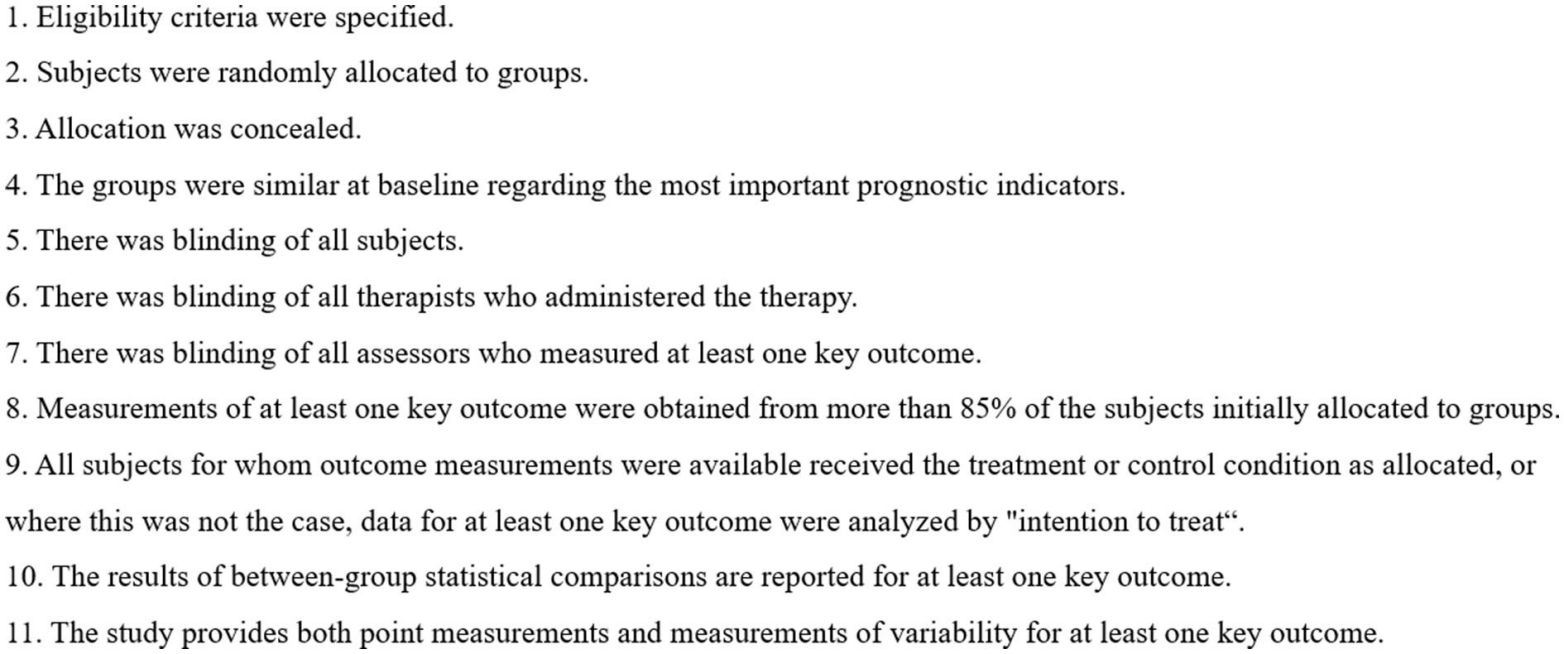
PEDro scale.

The Physiotherapy Evidence Database (PEDro) scale was used to evaluate the quality of each study (Maher et al., 2003) (Figure 2). Studies with a PEDro score <6 were considered to be of low quality. Each study's risk of bias was evaluated by using the Review Manager 5.4, which is based on the Cochrane Handbook for Systematic Reviews of Interventions (Cumpston et al., 2019). The risk of bias for each study was determined as “low risk,” “high risk,” or “unclear risk.” Researchers independently assessed the PEDro score and risk of bias of each study. If disagreements remained, a second researcher was consulted to establish a final consensus.

## Results

These databases yielded 142 related articles (43 in PubMed, 33 in EBSCO and 66 in Web of Science). After removing duplicate publications and identifying irrelevant studies by reviewing the titles, abstracts and full texts of the articles, 11 articles were included in this systematic review. These studies investigated the effects of tDCS on motor skills learning.

### Effects of tDCS on motor skills learning

Eleven studies investigated the effects of tDCS on motor skill learning. A total of 383 people were recruited (Table 1).

**Table 1:** Stimulation protocols and main outcomes of the studies investigating the effect of tDCS on motor skills learning.

**References**	**Sample**	**Age (year)**	**Anode Locations**	**Cathode Locations**	**Current (mA)**	**Electrode size (cm)**	**Current Density (mA/cm^2^)**	**Duration (min)**	**Task**	**Outcome measure**
Tecchio et al. (2010)	47	24-34	R M1	R Arm	1	7 × 5	0.03	15	SFTT	RTs for correct trials↓
Ferrucci et al. (2013)	21	20-49	R Cerebellar	R Arm	2	7 × 5	0.06	20	SRTT	RTs for correct trials ↓
Marquez et al. (2013)	30	20-27	R M1	R Arm	1	5 × 5	0.04	20	SFTT	RTs for correct trials↓
Cantarero et al. (2015)	33	24-31	R Cerebellar	R Buccinator	2	5 × 5	0.08	20	SVIPT	RTs for correct trials ↓
Zhu et al. (2015)	27	18-24	R Supraorbital	L DLPFC	1.5	5 × 5	0.06	15-20	Golf putting task	Number of successful putts ↑
Soekadar et al. (2015)	40	18-56	L M1	R Supraorbital	1	4 × 6	0.04	15	SRTT	RTs for correct trials ↓
Apolinario-Souza et al. (2016)	32	18-35	L M1	R Supraorbital	1	5 × 5	0.04	20	SFTT	RTs for correct trials ↓
Wessel et al. (2016)	38	20-28	R Cerebellar	R Buccinator	2	5 × 5	0.08	20	SFTT	RTs for correct trials ↓
Debarnot et al. (2019)	48	20-27	L M1	L Supraorbital	2	5 × 5	0.08	13	SRTT	RTs for correct trials ↓
Parma et al. (2021)	48	18-40	L M1	R M1	1.5	5 × 5	0.06	20	Golf putting task	Number of successful putts↑
Nakashima et al. (2021)	19	21-37	L DLPFC	R Forehead	2	7 × 5	0.06	20	SRTT	RTs for correct trials↓

R, right; L, left; M1, primary Motor Cortex; DLPFC, dorsolateral prefrontal cortex; RT, reaction time; SRTT, serial reaction time task; SFTT, sequential finger tapping tasks; SVIPT, sequential visual isometric pinch task. ↑ Means increase and ↓ means decrease.

**Figure 3:**
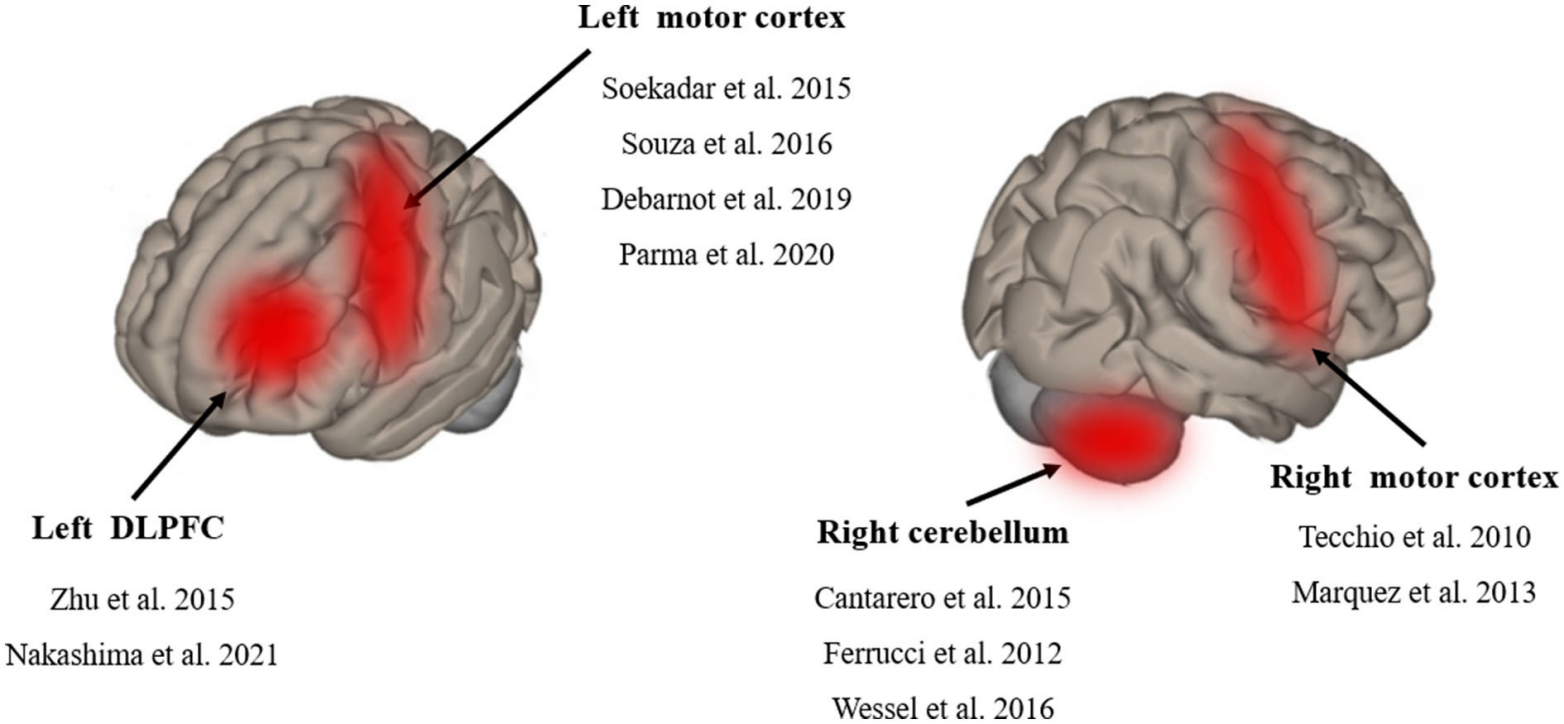
Schematic diagram of tDCS stimulation region.

Among the studies, four studies applied tDCS on the left M1 region (Soekadar et al., 2015; Apolinario-Souza et al., 2016; Debarnot et al., 2019; Parma et al., 2021). Two studies applied tDCS on the right M1 region (Tecchio et al., 2010; Marquez et al., 2013). Three studies applied tDCS on the right cerebellum (Ferrucci et al., 2013; Cantarero et al., 2015; Wessel et al., 2016) and two studies applied tDCS on the left DLPFC region (Zhu et al., 2015; Nakashima et al., 2021) (Figure 3). The current intensity of four studies was 1 mA (Tecchio et al., 2010; Marquez et al., 2013; Soekadar et al., 2015; Apolinario-Souza et al., 2016). The current intensity of five studies was 2 mA and that of two studies was 1.5 mA (Zhu et al., 2015; Parma et al., 2021). The electrode size in seven studies was 25 cm^2^ (Marquez et al., 2013; Cantarero et al., 2015; Zhu et al., 2015; Apolinario-Souza et al., 2016; Wessel et al., 2016; Debarnot et al., 2019; Parma et al., 2021). The electrode size in three studies was 35 cm^2^ (Tecchio et al., 2010; Kantak et al., 2012; Nakashima et al., 2021) and that in one study was 24 cm^2^ (Soekadar et al., 2015). The current density of 11 studies ranged from 0.03 to 0.08 mA/ cm^2^. The duration of stimulation in 10 studies was 15 to 20 min (Tecchio et al., 2010; Ferrucci et al., 2013; Marquez et al., 2013; Cantarero et al., 2015; Soekadar et al., 2015; Zhu et al., 2015; Apolinario-Souza et al., 2016; Wessel et al., 2016; Nakashima et al., 2021; Parma et al., 2021) and that in one study was 13 min (Debarnot et al., 2019). The number of montages for all studies was two electrodes.

At present, the most frequently tasks of investigating motor skills learning in the experimental environment are SRTT, SFTT and SVIPT (see Table 2 for details). Tecchio et al. (2010), Marquez et al. (2013), Soekadar et al. (2015), Apolinario-Souza et al. (2016) and Debarnot et al. (2019) found that compared with the sham condition, a-tDCS acting on the left and right M1 region could reduce the RTs for correct trials. Parma et al. (2021) reported that compared with the sham condition, a-tDCS acting on the left M1 region could increase golf putting performance. Ferrucci et al. (2013), Cantarero et al. (2015) and Wessel et al. (2016) discovered that compared with the sham condition, a-tDCS acting on the right cerebellar region could reduce the RTs for correct trials. Nakashima et al. (2021) showed that compared with the sham condition, a-tDCS acting the left DLPFC region also could reduce the RTs for correct trials. Meanwhile, Zhu et al. (2015) reported that compared with the sham condition, c-tDCS acting on the left DLPFC region also could improve golf putting performance. These phenomena demonstrated that increasing the excitability of a region involved in action observation promoted motor skills acquisition, and the results differed under the stimulation from different electrode polarities for the same region.

**Table 2:** Characterization of the main motor paradigms described in this Systematic Review.

**Motor sequence task**	**Description**
Serial Reaction Time Task (SRTT)	Participants responded to visual cues presented on the screen by pressing the relevant keyboard response. The location of visual cues was either presented in repeated sequences or at random.
Sequential Finger Tapping Tasks (SFTT)	A sequence of elements in a specific order that presented a specific finger movement was presented on the screen. Participants were required to perform the representative key operations as quickly and accurately as possible.
Sequential Visual Isometric Pinch Task (SVIPT)	Participants used their thumb and index finger to squeeze an isometric force sensor to control the movement of the pointer on the computer screen. The goal is to move the cursor as rapidly and correctly as possible between the beginning point and the target area's numbered sequence.

### Risk of bias and quality of evidence

**Figure 4:**
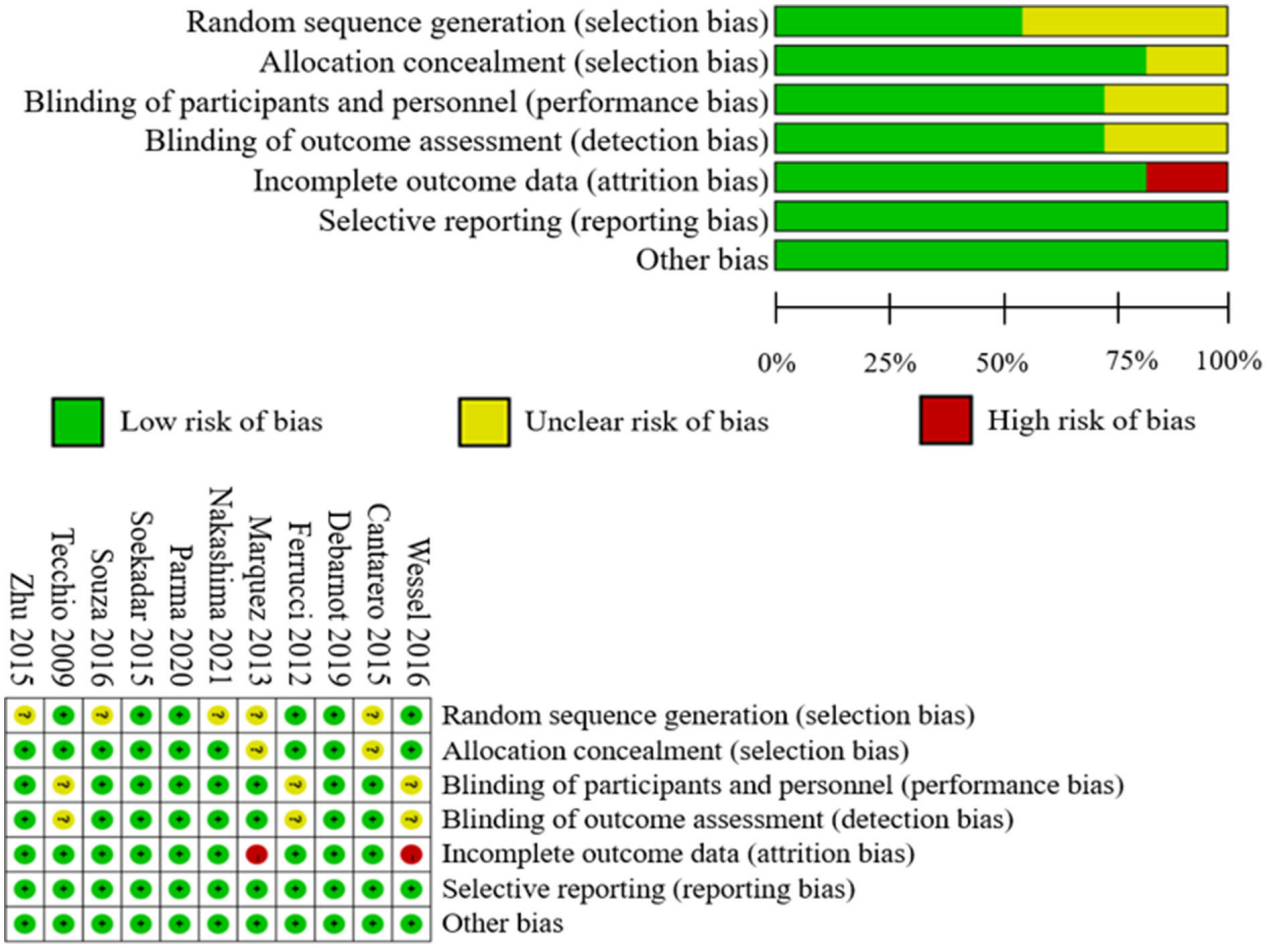
Summary of risk-of-bias assessment.

Figure 4 provides the summary risk of bias graph. In the risk-of-bias assessment, three studies maintained a low risk of bias in all domains tested, whereas the other studies revealed a certain high or unclear risk. All studies used randomization for random sequence generation. Six studies applied double blinding, and three studies utilized single blinding. Only two studies presented a high risk of incomplete outcome data. Complete data were not collected from one individual due to vertigo under stimulation. One sub-ject in the main experiment had to be excluded from the study due to vertigo under stimulation.

Two participants dropped out before the end of one study, resulting in incomplete data results (Marquez et al., 2013). All studies were evaluated to have a minimal risk of bias in terms of selective reporting. In addition, the PEDro score of all the studies exceeded 6, indicating that all of the included studies were of excellent quality. In a word, the investigations performed well in terms of allocation, blinding effectiveness, selective reporting, order effects avoidance, well-tolerated stimulation maintenance, and absence of side effects.

## Discussion

This study comprehensively evaluated the literature on the effects of tDCS on motor skills learning. The 11 included studies all demonstrated that tDCS can increase motor skills learning in healthy individuals by activating multiple brain regions, such as M1, left DLPFC and right cerebellum. However, owing to the small sample sizes, different tDCS parameters and other factors of the studies, these findings need to be validated and confirmed in future investigations. In the review, the underlying neurophysiological mechanism of tDCS were explored and future research directions were speculated.

### Influence of different electrode polarities on tDCS-induced motor skills learning and its possible explanation

The benefits of tDCS in increasing physical performance has been gradually studied in recent years (Lattari et al., 2020). These benefits include delaying muscle fatigue, increasing muscle strength, promoting motor skills learning and improving motor sensation (Machado et al., 2019).

Since Nitsche and Paulus (2000) reported the impact of transcranial low current on human M1 region, excitatory/inhibitory effects have been widely associated with anodal/cathodal current stimulation, respectively (Nitsche and Paulus, 2000). A-tDCS usually aims to depolarize the neuronal membrane potential to increase excitability in the target region, and the c-tDCS aims to hyperpolarize the neuronal membrane potential to inhibit excitability in the target region. Moreover, it should be pay attention that neuronal morphology (Nitsche and Paulus, 2000; Radman et al., 2009) and axonal orientation may be factors worth being considered when interpreting tDCS induced responses. Notably, the location of cathode and anode can also affect the differences in tDCS results. For example, Zhu et al. (2015) found that c-tDCS improved subjects' golf putting performance when the left DLPFC was stimulated by tDCS. However, Nakashima et al. (2021) found that a-tDCS was effective. Specifically, Zhu et al. (2015) placed the cathode contact on the subject's left DLPFC region and the anode contact on the right supraorbital region. While Nakashima et al. (2021) placed the anode on the subject's left DLPFC and the cathode on the right forehead. Although the current densities were the same in both studies, differences in the placement of the cathode and anode positions may have contributed to the differences in results.

Therefore, the differences in experimental tDCS results may be related to the high variability amongst individuals in terms of local circuit organization, basic functional levels, mental states, neurotransmitter levels, baseline neurophysiological states and genetic aspects (Machado et al., 2019). In addition, electrode shape and the montage (which includes the currents, locations and polarity of the electrodes), the stimulated brain region, and the brain state among other parameters can also influence the stimulation effect. Further research is needed to verify the effect of these factors on the stimulation of tDCS.

### Influences of different brain regions on tDCS-induced motor skills learning

Motor skills learning is a process wherein human body receives various signal stimuli and establishes complex conditioned reflex under the guidance of the cerebral cortex, and through practice to improve the ability of motor movement. The learning and adaptation of motor skills are associated with functional and structural changes in the brain distribution network including M1, somatosensory (S1), dorsal (PMd) and ventral premotor (PMv), SMA and posterior parietal cortex (PPC), as well as the cerebellum and basal ganglia (Landi et al., 2011). Stimulating brain regions associated with motor skills learning may have the same effects. The stimulating parameters for promoting motor skills learning are relatively variable. For example, most studies used the duration of 15 min to 20 min, and the polar plate is mostly 25 cm^2^. Previous works utilized commonly used valence motor skills learning tasks, including SRTT, SFTT and SVIPT (Ma et al., 2021). However, when these indicators were applied to evaluate motor skills learning, the task evaluation setting was relatively simple and ignored multidimensional and complex motor skills. Therefore, the improvement brought by tDCS was less than that expected. However, the main results of these studies suggested that tDCS has the potential to improve motor skills learning, and its effects should be further confirmed in future studies with increased sample sizes and rigors.

The motor cortex of the brain, involved in the execution and regulation of movement, it is mainly composed of three parts including M1 region, SMA region and premotor area (PMA). In view of the complexity involved in motor skills, multiple regions of the brain may be involved in the regulation and limitation of motor skills, including M1, DLPFC, SMA and the cerebellum (Machado et al., 2019). In addition, their basic principles may be different, so most studies on motor skills learning do not provide clear assumptions. For example, the reason placing electrodes at specific locations in the brain to stimulate or inhibit a given brain region can improve motor skills is unclear.

M1 region, the key brain region regulating human and animal motor execution, memory formation and motor skills consolidation, is the most associated region with motor skills and widely used in brain science (Fritsch et al., 2010). The M1 region has been found to be the main brain region stimulated by tDCS to improve motor skills learning and acquisition. tDCS is used on the M1 region to increase its excitability, which may lead to the continuous neural drive of neurons. In addition, under the condition of a-tDCS, the axons direction of the M1 region is perpendicular to the electrode surface, which can improve the excitability of neurons. M1 regulates the ability of the fingers to perform skilled, complex motor sequences (Rathelot and Strick, 2009), such as finger tapping tasks (Yu and Tomonaga, 2015). The action of a-tDCS on the M1 region can significantly increase the functional connection of the premotor, motor and sensorimotor regions of the cerebral hemisphere and induce the connectivity between the left and right hemispheres to change (Yang et al., 2021). Therefore, tDCS regulates the functional connection between the complex networks of the brain. tDCS can cause changes in the substances such as neurotransmitters, through a neuroplasticity model, which is based on the principle that current intensity leads to alterations in neuronal synapses and that local neuronal transmission causes the enhancement or weakening of synaptic transmission efficiency (Nwaroh et al., 2020). Glutamate (Glu) and γ-aminobutyric acid (GABA) are the two main neurotransmitters. Glu is an excitatory neurotransmitter, and GABA is an inhibitory neurotransmitter. Glu plays a key role in the maintenance of the synapses plasticity and promotes learning and memory by changing the efficacy of synaptic transmission and efficacy of the formation and function of cytoskeleton. Studies have shown that during or after tDCS, the levels of metabolites changed, such as increased excitatory neurotransmitters Glu and decreased inhibitory neurotransmitters GABA, especially in the cortical regions (Nwaroh et al., 2020). GABA plays an important regulatory role in skills learning. Therefore, the action of a-tDCS on the left M1 region could improve motor skills learning (Parma et al., 2021). In addition, some researchers believe that tDCS acted on M1 region affects motor performance by improving long-term changes in brain excitability and activity, through enhancing synaptic plasticity.

DLPFC is another region of interest that has always been a key candidate for working memory and interference control (Machado et al., 2019). Contemporary motor skills learning theory holds that motor learning can be obtained explicitly or implicitly (Zhu et al., 2015). Explicit motor learning is the language analysis aspect of learning through working memory management (Maxwell et al., 2003). By contrast, implicit motor learning reduces the involvement of language analysis in motor control by encouraging limited dependence on working memory. Compared with explicit motor learning, this form of learning has been proven to reduce awareness of the movements involved and increase neural efficiency (Zhu et al., 2015). A study showed that the DLPFC appears to be important to the neural basis of implicit motor learning, because the performance of patients with DLPFC lesions in practice tasks did not improve while the low-frequency repetitive transcranial magnetic stimulation (TMS) on the DLPFC improved motor learning, which suggested that DLPFC was a part of the neuronal matrix responsible for sequence learning (Hosp et al., 2011). Meanwhile, the action of tDCS on the left DLPFC demonstrated its clinical application in the treatment of depressive states, psychiatric symptoms, and the rehabilitation of cerebral infarction (Santos Ferreira et al., 2019). Zhu et al. (2015) and Nakashima et al. (2021) found that applying tDCS on the left DLPFC could improve implicit motor learning behavior (Zhu et al., 2015; Nakashima et al., 2021). In addition, Nakashima et al. (2021) found that a-tDCS improved implicit motor learning. However, Zhu et al. (2015) reported that c-tDCS improved implicit motor learning. Although the two studies had a consistent region of action, their tDCS intervention parameters were not completely consistent and their motor skills measures were inconsistent, which may account for the different results.

Cerebellum, an important motor regulation center, contributes to the regulation of programmed motor learning and plays important roles in establishing motor skills, perception, and motor behavior (Ehsani et al., 2016). The cerebellum helps control motor and non-motor behaviors, including learning, posture and balance, coordination, cognition, emotion, and language (Caligiore et al., 2017). The cerebellum is considered to be an alternative site to the M1 region for tDCS stimulation to promote motor learning (Oldrati and Schutter, 2018). In the process of motor learning, the cerebellum appears to play a crucial role in reducing errors associated with the needs of a new environment (Galea et al., 2011). The forming of motor skills is encoded by a large subcortical network that primarily involves the cerebellum (Ramnani, 2006). Clinical studies have shown that patients with damaged cerebellum have significantly impaired ability to learn new motor skills and poor ability to adapt to new environments (Criscimagna-Hemminger et al., 2010). These results revealed that the cerebellum was essential to the feedforward process required for motor adaptation. Ferrucci et al. (2013) and Cantarero et al. (2015) found that tDCS enhanced motor skills when it acted on the cerebellum, which was likely due to the increased excitability of cerebellar neurons, significant changes in the neural activity of interconnected parietal lobe network of the brain and the enhanced temporal complexity of distributed brain networks that modulated neural activity in interconnected cortical regions (Rastogi et al., 2017), thus improving motor skills.

### The disadvantages of tDCS, and the advantages of other non-invasive neuromodulation techniques

As a type of transcranial electrical stimulation, tDCS has defects. For example, it has a narrow action range and low spatial resolution, which make accurately stimulating deep brain regions difficult. Differences in electrode size, current parameters, stimulation duration and electrode position in different studies often have other adverse effects on the results (Rampersad et al., 2019). Meanwhile, the focus of the stimulating electric field itself is low, which is mainly manifested by the reduction in the intensity of the electromagnetic field with the increase in the distance from the head surface. Furthermore, numerous regions in the superficial cortex are directly involved in the body's cognition and behavior and participate in the whole brain (Bikson and Dmochowski, 2020; Lee et al., 2020). The network connection between tDCS stimulation regions and their inability to focus on deep brain regions may adversely affect the final results. Given that different regions of the brain may be related to different motor learning processes, the simultaneous electrical stimulation of these regions with the proper polarity and current intensity may optimize the relevant effects of tDCS. In this regard, bilateral M1 combined with PFC stimulation has been successfully applied. However, due to the inherent low focus of this technique, only a few effects related to its concomitant stimulation of different brain regions have been described in the literature. In the future, new non-invasive brain stimulation methods were needed to investigate its prospect in improving motor skills. The section on TI has been removed.

### Shortages of the review and prospect of tDCS research

The included studies generally showed that tDCS can promote motor skills learning. However, the results of this systematic review should be interpreted with caution given that the included studies had some methodological limitations. Firstly, the research sample size included in this review was relatively small. Secondly, the selection of tDCS parameters in different studies was diverse. The diversification of parameters led to diversified results. Therefore, a standardized tDCS scheme, a research design with increased rigor and advanced neural modeling technology for testing the effectiveness of tDCS in motor skills learning are urgently needed in future studies. Thirdly, this review only focuses on the effect of tDCS on motor skills learning in healthy adults. We need to further focus on exploring new directions for the treatment of neurological diseases and strengthen restorative treatments after stroke or other injuries affecting motor function.

The neurophysiological mechanisms of tDCS in motor skills are still unclear, and further researches are needed to estimate the target of tDCS by using neural modeling techniques combined with the participants' brain magnetic resonance images to correlate doses with the observed functional improvement. Notably, gender bias was found in the effect of tDCS on motor skills learning, but no research until now has reported how gender affects the effects of tDCS on motor skills, and this topic is also worth exploring in the future. In addition, the publication of negative results on the efficacy of tDCS can be encouraged to critique the implementation of tDCS for the potential publication bias, and such criticisms will eventually help optimize the tDCS intervention scheme.

In brief, although the mechanisms through which tDCS improves motor skills learning remains uncertain, several possible explanations exist and reflected in the change of cerebral excitability, the regulation of neurotransmitters, the increase in synaptic plasticity, the change in regional cerebral blood flow (rCBF) in the brain and the adjustment of brain network functional connections to regulate brain function (Bandeira et al., 2021). Improving cortical excitability and enhancing synaptic plasticity in target brain regions play key roles in regulating neural circuits (Nitsche et al., 2008). Specifically, the current delivered by tDCS may enhance synaptic connections between cortical neuronal structures, resulting in continuous changes in neural activity that can increase the degree of the synchronous discharge of motor units and further affect neural circuit regulation in motor skills and improve body functions (Patel et al., 2019).

## Conclusion

The existing research show that tDCS acted on multiple brain regions (including M1, DLPFC and cerebellum) can help improve motor skills learning, and its underlying neurophysiological mechanisms are related to the changes in cerebral excitability, neurotransmitters, synaptic plasticity, and brain network functional connections. However, future studies with large sample sizes and experimental designs with increased rigor are needed to confirm the current findings.

## Data availability statement

The original contributions presented in the study are included in the article/Supplementary material, further inquiries can be directed to the corresponding authors.

## Author contributions

SQ contributed to study design, data collection, drafting, and revising the manuscript. YL and XW contributed to supervising study design, completing data analysis and interpretation, and revising the manuscript. ZL and ZW revised the manuscript. All authors have read and approved the final version of the manuscript and agree with the order of the presentation of the authors.

## Funding

This work was supported by a grant from the key program of Natural Science Foundation of China (No. 11932013).

## Conflict of interest

The authors declare that the research was conducted in the absence of any commercial or financial relationships that could be construed as a potential conflict of interest.

## Publisher's note

All claims expressed in this article are solely those of the authors and do not necessarily represent those of their affiliated organizations, or those of the publisher, the editors and the reviewers. Any product that may be evaluated in this article, or claim that may be made by its manufacturer, is not guaranteed or endorsed by the publisher.
